# Regulation of Tomato Fruit Autophagic Flux and Promotion of Fruit Ripening by the Autophagy-Related Gene *SlATG8f*

**DOI:** 10.3390/plants12183339

**Published:** 2023-09-21

**Authors:** Cen Wen, Taimin Luo, Zhuo He, Yunzhou Li, Jianmin Yan, Wen Xu

**Affiliations:** 1 College of Agriculture, Guizhou University, Guizhou 550025, China; cenwen1098@163.com (C.W.); taiminl1296@163.com (T.L.); zhuohe0707@163.com (Z.H.); yzli1@gzu.edu.cn (Y.L.);; 2 Xingyi City Bureau of Agriculture and Rural Development, Guizhou 562400, China; 3 Guizhou Higher Education Facility Vegetable Engineering Research Center, Guizhou 550025, China; 4 Zhejiang Provincial Key Laboratory of Horticultural Plant Integrative Biology, Zhejiang University, Hangzhou 310058, China

**Keywords:** tomato, fruit ripening, autophagosome, autophagic flux, *SlATG8f*

## Abstract

Autophagy is a highly conserved self-degradation process that involves the degradation and recycling of cellular components and organelles. Although the involvement of autophagy in metabolic changes during fruit ripening has been preliminarily demonstrated, the variations in autophagic flux and specific functional roles in tomato fruit ripening remain to be elucidated. In this study, we analyzed the variations in autophagic flux during tomato fruit ripening. The results revealed differential expression of the *SlATG8* family members during tomato fruit ripening. Transmission electron microscopy observations and dansylcadaverine (MDC) staining confirmed the presence of autophagy at the cellular level in tomato fruits. Furthermore, the overexpression of *SlATG8f* induced the formation of autophagosomes, increased autophagic flux within tomato fruits, and effectively enhanced the expression of ATG8 proteins during the color-transition phase of fruit ripening, thus promoting tomato fruit maturation. *SlATG8f* overexpression also led to the accumulation of vitamin C (VC) and soluble solids while reducing acidity in the fruit. Collectively, our findings highlight the pivotal role of *SlATG8f* in enhancing tomato fruit ripening, providing insights into the mechanistic involvement of autophagy in this process. This research contributes to a better understanding of the key factors that regulate tomato fruit quality and offers a theoretical basis for tomato variety improvement.

## 1. Introduction

Autophagy is a highly conserved intracellular self-degradation system in eukaryotic organisms that is utilized to eliminate and recycle cellular components, thereby breaking down toxic substances or damaged organelles and reclaiming essential nutrients [1,2]. Currently, three types of autophagy have been mainly identified in plants: microautophagy, macroautophagy (referred to as “autophagy” hereafter), and mega-autophagy [2]. Among them, autophagy is the most crucial and prevalent form in plants, involving the encapsulation of cellular materials within double-membrane structures known as autophagosomes. Subsequently, the outer membrane of the autophagosome fuses with a vacuolar membrane, leading to the transfer of cellular contents to the vacuole where they are degraded by vacuolar proteases and hydrolases [3].

The formation of autophagosomes is governed by a plethora of autophagy-related genes (*ATGs*). Initially discovered in yeast [4], homologous *ATGs* have been subsequently identified in animals and plants. The proteins encoded by *ATGs* participate in various stages of the autophagy process [5]. Among the numerous ATG proteins, the ubiquitin-like protein ATG8 plays a pivotal role in autophagy. It is involved in autophagosome formation, membrane elongation and fusion, degradation of cellular contents, and energy release [6]. ATG8 protein binds to phosphatidylethanolamine in a ubiquitin-like conjugation reaction, serving as a marker for growing autophagosomes and complete autophagic bodies. On the one hand, the loss of *ATG8* function prevents autophagosome formation in yeast and other fungi [7]; however, this phenotype has not been observed in plants, which may be due to *ATG8* gene redundancy. On the other hand, the overexpression of *ATG8* promotes autophagosome formation in *Arabidopsis* and rice [8]. In addition to ATG8 lipidation, ATG8 protein is deaffixed by ATG4 and released from the autophagosome membrane, and the resulting released ATG8 protein is recycled to promote autophagosome formation in plants [9]. Consequently, ATG8 protein is often utilized as a reliable marker to assess autophagy induction and progression [10,11].

Research has indicated the significant role of autophagy in fruit ripening [9]. Transcriptome analysis of grape skin revealed elevated expression of *ATG18g*, *ATG9*, *ATG11*, and *ATG2* during the late stages of grape maturation [12], implying the involvement of autophagy-related genes in the regulation of grape fruit aging. Subsequently, López-Vidal et al. investigated autophagy (*ATG*) components, *ATG4* activity, autophagy receptor *NBR1*, protease *Lon1*, and protease *Lon2* gene expression and protein content changes in two pepper varieties. Their findings confirmed the presence of autophagic structures in pepper fruit, highlighting their crucial role in fruit maturation and quality formation [13]. Similarly, Sánchez-Sevilla et al. identified autophagy-related structures at the cellular level in different stages of strawberry fruit ripening and demonstrated that blocking autophagy significantly impairs strawberry fruit growth and maturation [14]. These studies underscore the importance of autophagy in plant fruit development, yet the variations in autophagic flux during fruit ripening and its associated functional roles remain to be elucidated.

Tomato (*Solanum lycopersicum* L.), one of the most esteemed vegetables globally, is renowned for its diverse flavors, forms, colors, and abundant nutritional value [15]. During tomato fruit ripening, the color transitions from green to red, the flesh softens, and the content compounds increase [16]. This process involves cell division, cell expansion, and a series of metabolic reactions [17]. With continuous technological advancements, the enhancement of tomato fruit quality has become a significant focus. Considering the synthesis and cycling metabolism of nutrients during fruit development, acquiring more information about the role of autophagy in this process is imperative. In this study, through differential expression analysis of autophagy-related gene members of the *ATG8* family during tomato fruit ripening, we demonstrate the existence of autophagic bodies within tomato fruit at the cellular level. Additionally, we observe the changes in ATG8 protein content necessary for autophagosome formation. Overexpression of *SlATG8f* leads to increased autophagic flux in tomato fruit, accelerating fruit ripening, upregulating ethylene-related genes, and enhancing fruit quality. Collectively, these findings suggest that autophagy is involved in regulating tomato fruit ripening and senescence, thereby enriching our understanding of the autophagic mechanisms in tomato fruit ripening and providing insights into the key factors that regulate fruit quality, ultimately contributing to the improvement of tomato varieties.

## 2. Results

### 2.1. Expression Analysis of SlATG8 Family Members during Tomato Fruit Ripening

**Figure 1:**
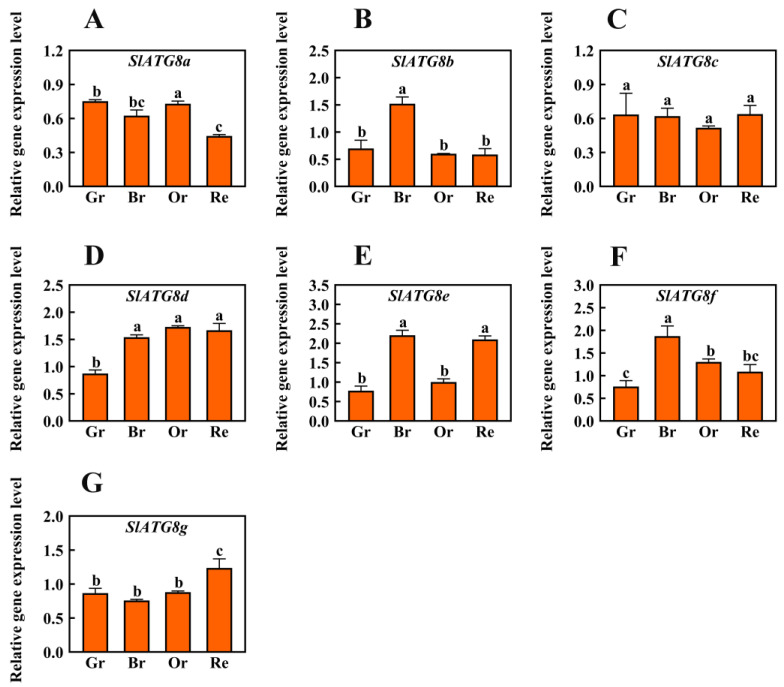
Differential Expression Analysis of *SlATG8s* During Tomato Fruit Ripening. The expression profiles of *SlATG8s* during different stages of tomato fruit ripening are shown. The stages of fruit ripening are as follows: green ripe (Day 30), breaker (Day 35), orange ripe (Day 40), and red ripe (Day 45). Abbreviations: Gr—Green Ripe; Br—Breaker; Or—Orange Ripe; Re—Red Ripe. (**A**–**G**)—*SlATG8a*, *SlATG8a*, *SlATG8b*, *SlATG8c*, *SlATG8d*, *SlATG8e*, *SlATG8f*, *SlATG8g* genes relative expression. The presented data represent the mean values, and error bars represent the standard deviation. The same letter indicates no significant difference in mean values at *p* < 0.05 (Duncan’s test).

In order to ascertain the involvement of autophagy in tomato fruit ripening, this study conducted an analysis of the expression levels of the core autophagy gene family, the *ATG8* family members. Quantitative real-time polymerase chain reaction (qRT-PCR) was employed to quantify the transcript levels of *SlATG8* family members during different stages of Micro-Tom tomato fruit maturation, including the green ripe, breaker, orange ripe, and red ripe stages (Figure 1). The results revealed distinct expression patterns among the *SlATG8* family members. During the ripening process of tomato fruits, *SlATG8a* and *SlATG8c* exhibited lower expression levels. In contrast, *SlATG8b* displayed elevated expression specifically during the color-breaking stage of tomato fruit ripening, while its expression was reduced during the green, orange, and red ripening stages. *SlATG8d* exhibited higher expression levels in the color-breaking, orange ripening, and red ripening stages compared to the green ripening stage. *SlATG8e* demonstrated significantly higher expression levels during both the color-breaking and red ripening stages, approximately twice as much as in the green ripening stage. Conversely, *SlATG8f* displayed its lowest expression level during the green ripening stage, peaked during the color-breaking stage, and decreased gradually from the color-breaking stage to the red ripening stage. Lastly, the expression of the *SlATG8g* gene was notably higher during the red ripening stage, lowest during the color-breaking stage, and relatively consistent during the green and orange ripening stages.

### 2.2. Overexpression of SlATG8f Promotes Pericarp Parenchyma Formation in Tomato Fruit

**Figure 2:**
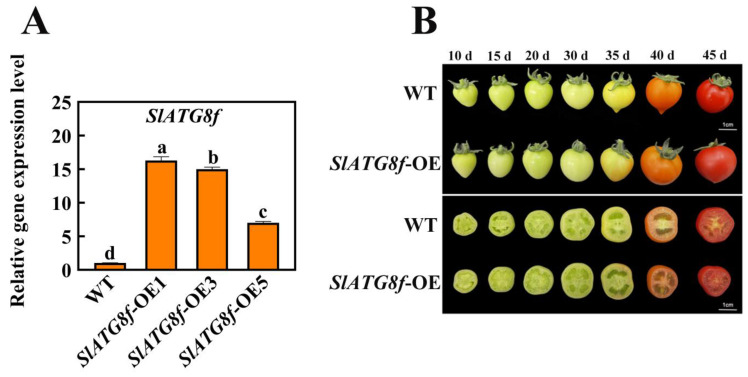
Identification of *SlATG8f* OE Plants Line and Phenotypic Observation of Fruits. (**A**) Quantitative real-time PCR (qRT-PCR) analysis of *SlATG8f* gene expression in the *SlATG8f* OE plants line compared to the wild-type (WT) plants. (**B**) Phenotypic observations and cross-sections at 10 days, 15 days, 20 days, 30 days, 35 days, 40 days, and 45 days after flowering. Abbreviations: WT—Wild Type; *SlATG8f*-OE—*SlATG8f* overexpressing plants. The data shown are the mean values of three replicates, and the error bars represent the standard deviation. The same letter indicates no significant difference in mean values at *p* < 0.05 (Duncan’s test).

In this study, transgenic Micro-Tom tomato plants were generated with overexpression of the *SlATG8f* coding sequence (CDS) under the control of the 35 S promoter. Quantitative real-time polymerase chain reaction (qRT-PCR) results demonstrated a substantial increase in *SlATG8f* gene expression in the *SlATG8f*-overexpressing (OE) plants, reaching up to 16-fold higher levels compared to the wild-type (WT) plants (Figure 2A).

Phenotypic changes in fruit development were observed among the different tomato plants during fruit ripening (Figure 2B). While there were no significant differences in fruit phenotypes between the *SlATG8f* OE plants and WT fruits, a notable dissimilarity was observed upon slicing the fruits. The pericarp parenchyma of the *SlATG8f* OE plants’ fruits exhibited accelerated development compared to that of the WT fruits with the pericarp parenchyma being more plump and filled in the *SlATG8f* OE plants’ fruits than in the WT fruits. In the early stages of fruit development, the pericarp parenchyma in the WT fruits was relatively scant, whereas the pericarp tissue of the *SlATG8f* OE plants’ fruits was already completely filled. By the 35th day of fruit development, both the *SlATG8f* OE plants and WT fruits had entered the liquefaction phase. However, the *SlATG8f* OE plants’ fruits displayed a higher degree of liquefaction in their pericarp parenchyma. In the late stages of fruit development, both the *SlATG8f* OE plants’ and WT fruits were fully matured, and their pericarp parenchyma had undergone significant liquefaction with no conspicuous differences between them. Nonetheless, the pericarp parenchyma tissue of the *SlATG8f* OE plants’ fruits remained fuller compared to that of the WT fruits.

### 2.3. Overexpression of SlATG8f Enhances Autophagic Activity in Tomato Fruit

**Figure 3:**
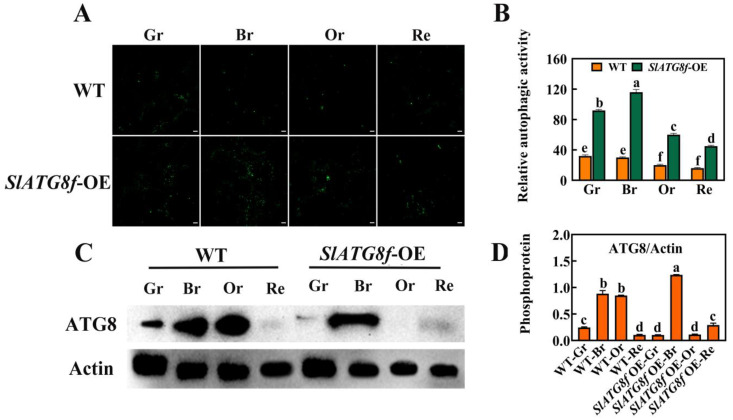
Effects of *SlATG8f* OE Plants on Autophagic Activity in Tomato Fruits. (**A**) Autophagosomes stained with MDC (green) in *SlATG8f* OE plants’ and WT fruits during different stages: green ripe (Gr), breaker (Br), orange ripe (Or), and red ripe (Re). Scale bars = 20 μm. (**B**) Relative autophagic activity quantified by counting the number of autophagosomes stained with MDC in each image from Panel A. (**C**) Western blot analysis of ATG8 protein expression in *SlATG8f* OE plants’ and WT fruits at different stages using Actin as a loading control. (**D**) Quantification of the intensity of each band from Panel C normalized to the intensity of the Actin band. ATG8/Actin ratio represents the relative expression of ATG8 protein. Abbreviations: white scale—20 μm; Gr—Green Ripe; Br—Breaker; Or—Orange Ripe; Re—Red Ripe; WT—Wild Type; *SlATG8f*-OE—*SlATG8f* overexpressing plants. The data shown are the mean values of three replicates, and the error bars represent the standard deviation. The same letter indicates no significant difference in mean values at *p* < 0.05 (Duncan’s test).

To investigate whether *SlATG8f* enhances autophagic activity in tomato fruit, this study employed MDC staining and protein immunoblotting (Western blotting) to assess the changes in autophagic activity between the *SlATG8f* OE plants’ and WT fruits at different stages of ripening (Figure 3). The MDC staining results revealed (Figure 3A,B) that, compared to the WT fruits, the *SlATG8f* OE plants’ fruits exhibited a higher intensity of green fluorescence corresponding to MDC-stained structures, indicative of autophagosomes. While the number of autophagosomes decreased from the green ripe stage to the red ripe stage in the WT fruits, the number of autophagosomes gradually increased from the green ripe stage to the breaker stage in the *SlATG8f* OE plants’ fruits, peaking at the breaker stage before declining.

Protein immunoblotting was employed to analyze the ATG8 protein levels during tomato fruit ripening, and the quantification of the corresponding bands was performed (Figure 3C,D). The results demonstrated that the ATG8 protein band intensity was strongest in the breaker stage of the *SlATG8f* OE plants’ fruits, whereas it was weakest in the green ripe, orange ripe, and red ripe stages, almost undetectable. In contrast, the ATG8 protein band signal was strong in the breaker and orange ripe stages of the WT fruits. The grayscale values of the extracted ATG8 protein bands indicated that, in the WT fruits, ATG8 protein expression gradually increased from the green ripe stage to the breaker stage followed by a decrease from the breaker stage to the orange ripe stage with the lowest expression in the red ripe stage. In the *SlATG8f* OE plants, ATG8 protein expression was significantly enhanced at the breaker stage, while it remained relatively low during the green ripe, orange ripe, and red ripe stages. These findings suggest that *SlATG8f* might be involved in the formation of autophagosomes during the breaker stage of tomato fruit.

**Figure 4:**
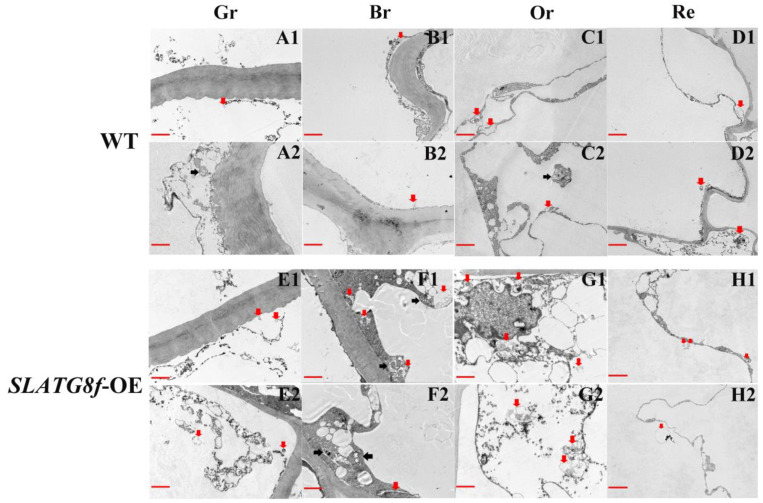
Effects of *SlATG8f* OE Plants on Autophagic Activity in Tomato Fruits. (**A1**–**D2**) Autophagic structures in WT tomato fruits at four stages of ripening. (**E1**–**H2**) Autophagic structures in *SlATG8f* OE plants’ tomato fruits at four stages of ripening. Scale bars = 2 μm. WT—Wild Type; *SlATG8f*-OE—*SlATG8f* overexpressing plants. Abbreviations: red scale—2 μm; Red arrows indicate autolysosomes (single-membrane structures with cytoplasmic components degraded), and black arrows indicate autophagosomes (double or multi-membrane vesicular structures containing cytoplasmic components). Gr—Green Ripe; Br—Breaker; Or—Orange Ripe; Re—Red Ripe; WT—Wild Type.

To gain deeper insights into whether the overexpression of *ATG8f* promotes an increase in autophagic flux during tomato fruit ripening, this study conducted an analysis of autophagic body structural changes using electron microscopy across four stages of fruit maturation (Figure 4). The results revealed distinct patterns of autophagic activity among the different fruit ripening stages. In the wild-type (WT) fruits, there was a relatively high number of autophagic bodies during the orange ripe stage, while autophagic activity was low in the other stages (Figure 4A–D). The *SlATG8f* OE plants’ fruits exhibited the highest autophagic activity during the breaker stage, displaying the highest number of visible autophagic bodies (Figure 4F). The following stage with notable autophagic activity was the orange ripe stage (Figure 4G), while autophagic activity was lower in the green ripe and red ripe stages (Figure 4E,H). Compared to the WT fruits, the *SlATG8f* OE plants’ fruits contained a greater abundance of autophagic structures within the cells, particularly evident during the breaker and orange ripe stages of the *SlATG8f* OE plants’ fruits. Notably, not only were numerous autolysosomal structures observed, but also, early autophagosome structures were evident (Figure 4F,G; indicated by double arrows and single arrows, respectively). These findings underscore that overexpression of *SlATG8f* significantly enhances autophagic flux during tomato fruit ripening.

### 2.4. Overexpression of SlATG8f Facilitates Tomato Fruit Ripening

**Figure 5:**
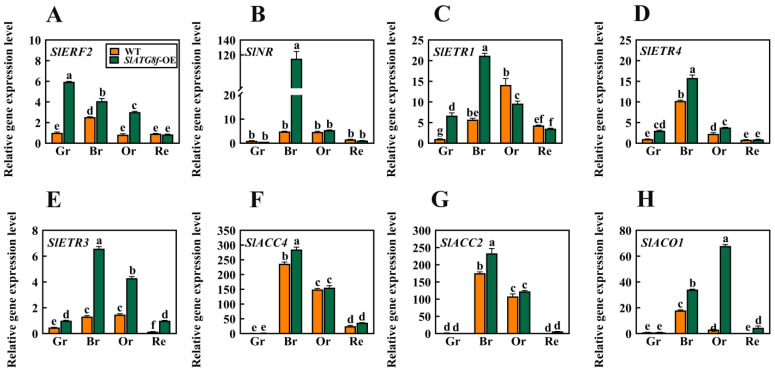
Effects of *SlATG8f* OE Plants on the Expression of Ethylene-Related Genes in Tomato Fruits. (**A**) qRT-PCR analysis of the expression of the ethylene signal transduction gene *ERF2*. (**B**–**E**) qRT-PCR analysis of the expression of ethylene receptor genes *NR*, *ETR1*, *ETR4*, and *ETR3*. (**F**–**H**) qRT-PCR analysis of the expression of ethylene synthesis genes *ACC4*, *ACO1*, and *ACC2*. Annotations: Gr—Green Ripe; Br—Breaker; Or—Orange Ripe; Re—Red Ripe; WT—Wild Type; *SlATG8f*-OE—*SlATG8f* overexpressing plants.The data shown are the mean values of three replicates, and the error bars represent the standard deviation. The same letter indicates no significant difference in mean values at *p* < 0.05 (Duncan’s test).

In earlier investigations, it was observed that the degree of liquefaction of the pericarp parenchyma in the *SlATG8f* OE plants was higher than that in the WT plants at the 35th day of fruit development. Based on this finding, this study hypothesized that *SlATG8f* might promote tomato fruit ripening. To validate this hypothesis, a selection of genes associated with ethylene signaling was screened, including ethylene signal transduction gene *ERF2*; ethylene receptor genes *NR*, *ETR1*, *ETR3*, and *ETR4*; and ethylene synthesis genes *ACC2*, *ACC4*, and *ACO1*. Their expression level changes during fruit ripening were analyzed (Figure 5).

The results indicated that, in the *SlATG8f* OE plants’ fruits, the expression of *SlERF2* was upregulated during the green ripe, breaker, and orange ripe stages (Figure 5A). *SlNR* was significantly upregulated during the breaker stage of the *SlATG8f* OE plants’ fruits (Figure 5B). The expression levels of *SlETR1* and *SlETR4* were elevated during the green ripe and breaker stages of the *SlATG8f* OE plants’ fruits, but their expression levels were not notably high during the orange ripe and red ripe stages (Figure 5C,D). *SlAETR3* exhibited increased expression during the breaker, orange ripe, and red ripe stages of the *SlATG8f* OE plants’ fruits (Figure 5E). *SlACO1* showed significant upregulation during the breaker and orange ripe stages of the *SlATG8f* OE plants’ fruits (Figure 5G). However, the expression levels of *SlACC4* and *SlACC2* during the breaker and orange ripe stages of the *SlATG8f* OE plants’ fruits did not differ significantly from those of the wild type (Figure 5F,H). These findings suggest that *SlATG8f* might influence ethylene production by regulating the expression of *SlACO1* and, thereby, promoting fruit ripening.

### 2.5. Overexpression of SlATG8f Improvements of Tomato Fruit Quality

**Figure 6:**
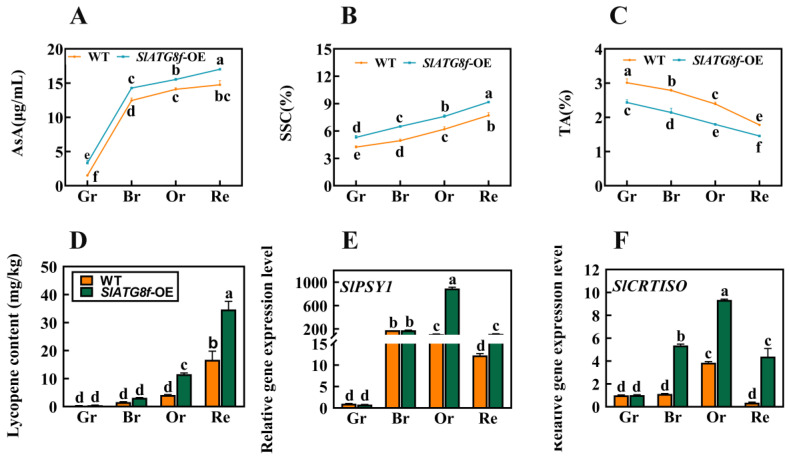
Effects of *SlATG8f* OE Plants on Pigment Accumulation, Soluble Solids, and Acidity in Tomato Fruits. (**A**) Ascorbic acid (AsA) content in tomato fruits. (**B**) Soluble solids (SSC) content in tomato fruits (expressed as a percentage). (**C**) Titratable acidity (TA) content in tomato fruits (expressed as a percentage). (**D**) Lycopene content in tomato fruits. (**E**,**F**) qRT-PCR analysis of the expression levels of lycopene synthesis genes *SlPSY1* and *SlCRTISO*. qRT-PCR primer sequences are provided in Supplementary Table S1. Annotations: Gr—Green Ripe; Br—Breaker; Or—Orange Ripe; Re—Red Ripe; WT—Wild Type; *SlATG8f*-OE—*SlATG8f* overexpressing plants. The data shown are the mean values of three replicates, and the error bars represent the standard deviation. The same letter indicates no significant difference in mean values at *p* < 0.05 (Duncan’s test).

To further comprehend whether the promotion of fruit ripening by the *SlATG8f* OE plants influences fruit quality and flavor, this study evaluated the content of ascorbic acid (AsA), soluble solids content (SSC), titratable acidity (TA), and tomato lycopene levels. The results revealed notable variations in these components. At all ripening stages of the *SlATG8f* OE plants’ fruits, the content of both ascorbic acid and soluble solids was higher compared to that of the WT fruits (Figure 6A,B). Conversely, the titratable acidity content was consistently lower in the *SlATG8f* OE plants’ fruits compared to that in the WT (Figure 6C). During the orange ripe and red ripe stages of fruit development, the *SlATG8f* OE plants’ fruits exhibited significantly higher levels of tomato lycopene content compared to the WT fruits (Figure 6D). The key gene involved in tomato lycopene synthesis, *SlPSY1*, was notably upregulated during the orange ripe stage of the *SlATG8f* OE plants’ fruits (Figure 6E). Additionally, the expression of the tomato carotenoid isomerase gene, *SlCRTISO*, was elevated during the breaker, orange ripe, and red ripe stages of the *SlATG8f* OE plants’ fruits compared to that of the WT fruits (Figure 6F). These findings led to the hypothesis that overexpression of *SlATG8f* could enhance tomato fruit quality, potentially mediating the biosynthesis pathway of tomato lycopene and, thereby, influencing the color change in tomato fruits.

## 3. Discussion

Autophagy is a crucial process in eukaryotic cells for the degradation and selective elimination of cytoplasmic components, playing a significant role in the bulk and selective degradation of targeted substances [18]. In plant cells, autophagy involves the transfer of damaged or redundant macromolecules and organelles to vacuoles for degradation, which holds key roles in maintaining cellular homeostasis, growth, and development as well as responding to environmental stress [19]. However, the role of autophagy in fruit development and ripening remains largely unexplored, and the analysis of ATG genes or ATG proteins in this context is seldom reported. Bernard et al. suggested that the transcriptional upregulation of ATG genes enhances autophagy [20]. In our study, the expression of tomato *SlATG8s* was detected during fruit ripening, indicating the presence of autophagy activity in these fruits. *SlATG8b*, *SlATG8d*, *SlATG8e*, and *SlATG8f* genes exhibited higher expression levels during the breaker stage; *SlATG8d* gene showed higher expression during the orange ripe stage; and *SlATG8d*, *SlATG8e*, and *SlATG8g* genes showed higher expression during the red ripe stage. This suggests that these genes might activate distinct regulatory mechanisms at different stages of tomato fruit ripening. In Arabidopsis, nine *ATG8* homologous genes have been identified that encode proteins whose C-terminal cleavage is *ATG4*-dependent followed by the formation of ATG8-PE complexes [21]. ATG8-PE complexes promote the initiation of autophagosomes [22]. However, in our study, changes in the expression of ATG8 protein levels were detected using Western blotting, while the expression of ATG8-PE complexes was not assessed, suggesting the need for further investigation.

The presence of multiple autophagosome vesicles in tomato fruit vacuoles indicates the pivotal role of autophagy in fruit ripening. Through transmission electron microscopy, an increased number of vesicles was observed in the *SlATG8f* OE plants’ fruits compared to those in the WT with the peak vesicle count occurring during the turning stage, a result corroborated by MDC staining. As fruit undergoes various metabolic shifts during the transition from green to red, including the transformation of chloroplasts to chromoplasts [23], it suggests that different autophagy mechanisms might take place to sustain energy for fruit maturation. Prior research on peppers and strawberries indicated a higher autophagy activity in yellow peppers compared to that in red peppers as well as two strong autophagy fluxes during the white and ripe stages of strawberry maturation [12,14]. Our findings also suggest that the peak autophagy activity during the breaker and orange ripe stages of tomato fruit ripening, enhanced autophagy activity during the breaker stage in the *SlATG8f* OE plants’ genotype, is advantageous for energy balance during fruit maturation.

Autophagy primarily participates in plant aging and senescence [24]. The process of fruit ripening is also an aging process that is dependent on cellular vitality and gene regulation [25]. Among these, ethylene-related genes play pivotal roles in tomato fruit ripening [26]. Our research revealed increased liquefaction of the locule in the *SlATG8f* OE plants’ fruits during the breaker stage accompanied by upregulated expression of ethylene-related genes. This, in conjunction with the high expression of ATG8 protein during the breaker stage of the *SlATG8f* OE plants’ fruits, further confirms the promoting role of autophagy in tomato fruit ripening. Simultaneously, autophagy induced during fruit ripening enhances fruit coloration, increases levels of ascorbic acid and soluble solids, reduces acidity, and improves fruit texture and quality—possibly arising from the dual effect of autophagy. Previous studies reported that autophagy promotes strawberry fruit ripening while delaying senescence [27]. Our results suggest the presence of autophagy during tomato fruit ripening with the peak autophagy activity during the breaker and orange ripe stages; *SlATG8f* OE plants effectively enhance autophagy activity during the breaker stage, promoting fruit maturation and enhancing fruit quality. These findings have significant implications for understanding the role of autophagy in tomato fruit growth and development, exploring the regulatory mechanisms of autophagy in fruit, and advancing tomato breeding strategies.

## 4. Materials and Methods

### 4.1. Plant Materials and Growth Conditions

Micro-Tom tomato seeds were disinfected with 0.3% sodium hypochlorite for 10 min followed by three rinses with sterile water. After soaking in water at 55 °C for 15 min and then in water at 28 °C for 6 h, the seeds were germinated in darkness at 28 °C. Once the seeds were visibly germinated, they were sown in trays containing soilless growth medium. The growth chamber conditions were set at 18 h light/6 h dark with a constant temperature of 25 °C. Fruit samples were collected at different ripening stages: green ripe (day 30), breaker (day 35), orange ripe (day 40), and red ripe (day 45). For each stage, 4–5 fruits were sampled, and the middle portion of the peel and flesh was harvested, frozen in liquid nitrogen, and stored at −80 °C. Gr: green ripe; Br: breaker; Or: orange ripe; Re: red ripe.

### 4.2. Generation of Transgenic Plants

The full-length SlATG8f coding sequence (CDS) was amplified from tomato cDNA using the specific primer (Supplemental Table S2) into pBWA(V)HS and then introduced into the plant overexpression vector. The resulting pro35S, SlATG8f, was transformed into Agrobacterium tumefaciens strain GV3101 and then transformed into Micro-Tom tomato with Agrobacterium-mediated transformation. During tomato infection, the tomato seeds were first surface-sterilized with 75% alcohol and sodium hypochlorite solution followed by immersion cleaning in sterilized distilled water to obtain sterile materials. After that, the sterilized seeds were spread on 1/2 MS solid agar medium (pH = 5.8) and left to germinate, and the cotyledons were assessed for Agrobacterium infection. Kanamycin was used for selecting stable transformants based on their resistance. Finally, the T2 generation was chosen as the experimental material.

### 4.3. Quantitative Real-Time PCR (qRT-PCR)

Quantitative real-time PCR was employed to study the expression of SlATG8s and ethylene-related genes in tomato fruits. Frozen tomato fruit samples were used for RNA extraction with a TIANGEN kit (DP432, TIANGEN), and cDNA synthesis was performed using a reverse transcription kit (KR118, TIANGEN). Specific primers were designed using Primer 5.0 software with the Actin gene selected as the internal reference (Supplementary Table S1). qRT-PCR was conducted using a 7500 Real-Time PCR System (4351107, Thermo Fisher Scientific, Guangzhou, China) and SYBR Green Supermix (RK02001, Tiangen, Shanghai, China). The experiments were carried out with three biological replicates and three technical replicates.

### 4.4. Transmission Electron Microscopy Observation

Tomato fruits were soaked in a 100 nmol/L rapamycin solution for 12 h to induce autophagy. The fruit tissues were rapidly cut into 1–2 mm^3^ pieces, fixed in electron microscopy fixative solution (G1102, Servicebio, Wuhan, China) at 4 °C for 2–4 h, and then dehydrated using a series of acetone gradients. The dehydrated tissues were infiltrated and embedded in mixtures of dehydrating agents and epoxy resin in ratios of 3:1, 1:1, and 1:3. Ultrathin sections (50 nm) were obtained using an ultrathin slicer, and these sections were placed on copper grids. The sections were stained with uranyl acetate for 10–15 min and then with lead citrate for 1–2 min at room temperature. Transmission electron microscopy was performed using a JEM-1400PLUS (JEM-1400 plus, Jeol, Tokyo, Japan) instrument.

### 4.5. Dansylcadaverine (MDC) Staining

Tomato fruits were soaked in a 100 nmol/L rapamycin solution for 12 h to induce autophagy. The tomato fruit sections were prepared according to a previous protocol [28]. The sections were immediately stained with 100 μM MDC (30432, Sigma-Aldrich, Shanghai, China) under vacuum and dark conditions for 30 min and then washed twice with phosphate-buffered saline (PBS, P1020, Solarbio, Beijing, China). Autophagosome structures were observed using an LSM-780 confocal microscope (LSM-780, Carl Zeiss, Shanghai, China) with excitation at 405 nm and emission ranging from 400 to 580 nm.

### 4.6. Protein Blot (Western Blotting)

Tomato fruits were soaked in a 100 nmol/L rapamycin solution for 12 h to induce autophagy. The fruit tissues were frozen in liquid nitrogen and used for total protein extraction using a kit (PTE011, Coolaber, Beijing, China). SDS-PAGE gel electrophoresis was performed (P1200, Solarbio, Beijing, China) to separate the proteins. The membrane was blocked with 5% skim milk for 2 h and then washed with TBST. The PVDF membrane was cut into strips and incubated with primary antibodies (1:1000) overnight at 4 °C. After washing with TBST three times, the membrane was incubated with secondary antibodies (1:1000) against rabbit ATG8a polyclonal antibody (Ab77003, Abcam, Shanghai, China) for 1 h at room temperature. Chemiluminescence was detected using a chemiluminescence detection kit (34080, Thermo Fisher Scientific, Guangzhou, China).

### 4.7. Detection of Lycopene Content, Ascorbic Acid, Soluble Solids (SSC), and Titratable Acidity (TA)

The lycopene content of the tomato fruits was determined according to Sun et al.’s method. Five grams of tomato flesh and peel tissue were frozen and ground into powder. The powder was mixed with 50 mL of hexane–acetone–ethanol (2:1:1, *v*/*v*) and shaken. After adding 15 mL of water and allowing for phase separation, the organic phase (hexane) absorbance was measured at 503 nm to calculate the lycopene concentration. The molar extinction coefficient was 17.2 L mol^−1^·m^−1^, and the units were expressed as mg·kg^−1^ based on the fresh weight [29]. Ascorbic acid was measured using a kit (A009-1-1, Nanjing Jiancheng Bioengineering Institute, Nanjing, China). The SSC and TA content measurements were performed using a PAL-BX/ACD1 sugar acidity meter (ATAGO, Tokyo, Japan) for SSC (%) and TA (%) in the fruit. The experiments included three biological replicates and three technical replicates.

## 5. Conclusions

In this study, quantitative analysis of the expression of the *ATG8* gene family members during tomato fruit ripening revealed that the expression levels of most *ATG8* family members were higher during the fruit color transition period or late stages of fruit ripening. Among them, the expression of *SlATG8f* gradually increased from the green ripe stage to the breaker stage and then gradually decreased from the breaker stage to the red ripe stage. In the *SlATG8f* OE plants line, the locular gel tissue of the tomato fruits appeared more plump compared to that in the wild type (WT), and the liquefaction rate of the locular gel tissue was faster, indicating that the *SlATG8f* gene positively regulates tomato fruit development. The MDC staining and electron microscopy observations revealed that the number of autophagosomes in the *SlATG8f* OE plants’ fruits was significantly higher than that in the WT, especially during the breaker stage, with a substantial increase in the number of autophagosomes and autolysosomes in the fruit flesh cells, indicating enhanced autophagic activity. Western blot results demonstrated a significant increase in ATG8 protein expression during the breaker stage in the *SlATG8f* OE plants’ fruits, and the expression level of *SlATG8f* correlated positively with autophagic activity. Overexpression of *SlATG8f* induced the formation of autophagosomes in the tomato fruit cells, enhancing autophagic activity. The increased autophagic flux due to *SlATG8f* overexpression accelerated fruit ripening and upregulated the expression of ethylene-related genes, regulating fruit ripening. Taken together, our findings suggest that autophagy is involved in regulating tomato fruit ripening and senescence. These discoveries contribute to a deeper understanding of the role of autophagy in the process of tomato fruit ripening, elucidate the key factors that regulate fruit quality, and provide a theoretical basis for improving tomato varieties.

## Data Availability

Data are available in the manuscript and in the Supplementary Materials.

## Supplementary Materials

The following supporting information can be downloaded at: https://www.mdpi.com/article/10.3390/plants12183339/s1, Table S1: ATG8 family members, ethylene-related genes, *SlPSY1*, *SlCRTISO* primer design table, Table S2: SlATG8f clone-specific primers.
