# Supplementary material for: Regulation of Tomato Fruit Autophagic Flux and Promotion of Fruit Ripening by the Autophagy-Related Gene SlATG8f
Source: Plants (Basel). 2023 Sep 21;12(18):3339. doi: 10.3390/plants12183339 (PMC10536916; doi:10.3390/plants12183339)
Supplement: Supplementary file 1 [file plants-12-03339-s001.zip › plants-2589532-supplementary/Table S1.pdf]

Table S1. *ATG8* family members, ethylene-related genes, SlPSY1, SlCRTISO primer design table.

Gene ID	Gene	Primer sequence(5'→3')
Solyc01g068060	*SlATG8a-F*	CGTGAAGCAAAGAAATGGGGAAGAC
	SlATG8a-R	ACCGGCACACGATCAGGATATTTG
Solyc02g080590	*SlATG8b-F*	TTATGTGGTGCGGAAGAGGATCAAG
	SlATG8b-R	AGAGAAGCTGTTGGAGGCAAAGTG
Solyc03g031650	*SlATG8c-F*	TCCGAAAGAGGATCAATCTCAGTGC
	SlATG8c-R	CAGACATCAGAGCAGCAGTTGGAG
Solyc07g064680	*SlATG8d-F*	GCTGCCATGATGTCTGCCATTTATG
	SlATG8d-R	GAAGGTATTCTCGCCACTGTAGGTC
Solyc08g007400	*SlATG8e-F*	TTGACGGTAGGGCAGTTTGTGTATG
	SlATG8e-R	TGGAAGACATGATTGCACCTGTTGG
Solyc08g078820	*SlATG8f-F*	ACAATGTGCTACCGCCAACAGG
	SlATG8f-R	GGTAACATAGAGGAAGCCGTCATCG
Solyc10g006270	*SlATG8g-F*	TTGGTTCCTGCTGATCTAACTGTGG
	SlATG8g-R	TGCTCCTCATAAATGGCGGACATC
AY192368.1	*ERF2-F*	CGTTTGTCATCCACCGACCT
	ERF2-R	GTCACGAATTTCAGCAGCCC
U38666.1	*NR-F*	ATCGCATTCTCCGTCGTCAT
	NR-R	TCCATTATCTCGTTTCGTCCC
NM_001247220.2	*ETR1-F*	ATGGATGAGAATGGTGTTAGCAGGA
	ETR1-R	CACAATAAGTGGCCTACCGTGACGT
AF118843.1	*ETR4-F*	GTTCTTGGGCTTCAACTGCG
	ETR4-R	ACAGCAGGGCTAAGAACACC
NM_001247276.2	*ETR3-F*	AAGGGAACCACTGTCACGTTTGTAG
	ETR3-R	TTAATGTTCTTTGTCACACCAATGTCC
Solyc05g050010	*ACC4-F*	CAAGCACAATGGAAGAGGAACAACC
	ACC4-R	CGCACTACGAGCAAGGAATTGGAG
Solyc07g049530	*ACO1-F*	CCACCATGTCCTAAGCCCGATTTG
	ACO1-R	TTGCTCGTCTTTGAGGAGTTGAAGG
Solyc01g095080	*ACC2-F*	GCTTAACGTCTCGCCTGGATCTTC

	ACC2-R	CTCAACACCTACGAACCTCCGAATC
Solyc03g031860	*PSY1-F*	TTGCTGGAAGGGTGACCGATAAATG
	PSY1-R	ACAAGACCAAAGATGCCCATACAGG
AF416727	*CRTISO-F*	TTTTGGCGGAATCAACTACC
	CRTISO-R	GAAAGCTTCACTCCCACAGC
Solyc03g078400	*Actin-F*	TGGTCGGAATGGGACAGAAG
	Actin-R	CTCAGTCAGGAGAACAGGGT
