# Supplementary material for: Regulation of Tomato Fruit Autophagic Flux and Promotion of Fruit Ripening by the Autophagy-Related Gene SlATG8f
Source: Plants (Basel). 2023 Sep 21;12(18):3339. doi: 10.3390/plants12183339 (PMC10536916; doi:10.3390/plants12183339)
Supplement: Supplementary file 1 [file plants-12-03339-s001.zip › plants-2589532-supplementary/Table S2.pdf]

Table S2. *SlATG8f clone-specific primers*

Primer	Sequence（5’-3’）
Primer-F	cagtGGTCTCacaacatggctaagagctcattcaa
Primer-R	cagtGGTCTCatacacagttcgctcaggaccccga
